# The Action of Different Medicines Used in Dental Practice

**Published:** 1874-10

**Authors:** John Murray

**Affiliations:** Rochester, Pa.


### SELECTED ARTICLES.

---

### ARTICLE IV.

### *The Action of Different Medicines Used in Dental Practice.*

BY JOHN MURRAY, D. D. S., ROCHESTER, PA.

In attempting to perform the duty assigned to me at this hour, by your Executive Committee, it will be my effort to consider the remedies, or at least a few of the remedies employed by our profession.

If I rightly comprehend the work allotted to me, it is to the sources from which these remedies are derived, the mode of their preparation, their sensible properties, their chemical composition and their relations, their physiological effects, or the effects they are capable of producing in healthy individuals, and their modus operandi. Their therapeutical effects, or those which they produce in morbid states of the system, I shall leave to be discussed by my brother essayist who has that theme committed to his especial care.

To facilitate a uniform nomenclature and dispensation of medicines, authoritative works have been issued in London, Paris, Dublin and in the United States, termed Pharmacopœia. The Pharmacopœia of the United States was first published by the authority of a convention held at Washington in the year 1820, and it has since been revised every ten years. It furnishes a list of articles which are in general use, and supplies formulæ for such preparations as are kept in the shops, and which are thence termed offecinal, from the Latin word *offecina*, a shop.

The unbounded credulity which at one time prevailed regarding the effects of drugs, has now almost passed away, and we observe less and less of the old feeling of confidence in the adaptation of particular drugs to peculiar cases of disease. The plan now is, to discover the seat and nature of diseased action, and to adapt a remedy whose properties are known, to the exigency, locally or generally, as the case may require. We have no agents that are possessed of specific properties which are exerted with unerring certainty and uniformity on disease. Although we may be perfectly acquainted with the ordinary medicinal properties of a drug, and although these properties may be essentially the same, the agency exerted by it may be different according to the sex, temperament, &c., of the patient. Were these points determinate, we could always calculate with a certainty what would be the precise action of any medicinal agent. Medicines are capable of affecting every tissue and every function, directly or indirectly. It must be remembered that however faithfully and correctly the ordinary action of medicines may be set forth, the ordinary allowance must be made for surrounding circumstances.

Prior to entering upon the main feature of my subject—the action of medicines—it may not be considered out of place to spend a few moments on the modus operandi of medicines upon living subjects or substances.

There are two general methods by which the impressions of medicines are obtained: 1st. When it is desired that an impression shall be made at some remote part of the body, or when it is desired that it shall permeate every part the exhibition is internal. When a drug is taken into the stomach, it may merely affect that organ by simple contact. This is the simplest mode in which medicinal agents act. When a drug is permitted to remain as part of the contents of the stomach for any considerable length of time, other parts become impressed according to the elective affinity of the particular article for some tissue or organ rather than another. The precise mode of operation by which this is brought about has been a subject of controversy. All writers of modern date agree that this process is accomplished by the circulation. The absorbents take up the particles and pass them into the blood, and so transmit them to remote parts of the body. This is the method that is mainly relied upon by the general practitioner. There is another mode of using remedies, and one to our profession of equal if not greater importance and interest than the former—the topical or local application of remedies or medicines. If, in this mode of their application, we are denied the active system of absorbents, and the circulation to accomplish the desired end, we at least have the advantage of a direct and local application to the very spot desired to be reached. But while our dependence is principally upon the local or topical application of remedies, we hold to the opinion that the system is abundantly able to appropriate these local remedies to its local necessities. The experience that has been furnished by many years of practice and observation, has taught us, and the whole profession, that there are a limited nnmber of medicines which, topically applied, not only make a decided impression upon the immediate locality, but carried by capillary absorption or other agents, reach points remote from the place of application. "Headland states, that some few substances may act locally by irritation or otherwise, on the mucous surfaces of the stomach or intestines. These are not many; they act without being absorbed, and they may not extend into the system at large. In some few cases these local actions may be succeeded by changes in distant parts on the principle of revulsion." As the living body is supplied with a regular and perfect system of secretary organs by which every part is thoroughly relieved of every particle of effete matter, so nature has provided us with a no less perfect, efficient and universal system of absorbents, whose office it is to take up and appropriate whatever may be necessary to the sustenance and health of the individual. While the cuticle is destitute of a living organism, yet it is not incapable of imbibition, but is subject to the general law of endosmosis. May not the numerous orifices which open upon the cuticle perform the double office of secretion and absorption ? It is well known that persons suffering from excessive thirst will have it very much assuaged by partial immersion in water. This must be accounted for by the law of absorption or imbibition through the innumerable apertures of the skin. Now what has been asserted in reference to the powers and capabilities of the soft tissues to appropriate to their varied wants whatever may be within their reach, may also with equal propriety and confidence be asserted in regard to the hard tissues, and particularly of dentine. Dentine itself, considered as a living substance, and without any regard to its internal structure of vessels, or its sympathetic external surroundings of blood vessels, gums, nerves, &c., possesses a living organization that will justify the conclusion that through its numerous tubuli it will convey to its remotest portions in fluences that may be topically put within its reach. What the tooth cannot do of itself, in consequence of the low degree it holds in the scale of vitality, its pulp and system of nerves and blood vessels, will fully and perfectly accomplish. Who of us has not experienced the capability of a tooth to absorb arsenious acid to its own destruction and that of its adjacent tooth or teeth.

With these preliminary remarks on general principles connected more or less intimately with the action of medicines that are the most extensively employed in the practice of our profession.

Prominent in the list of the Dental Materia stands Creosote first to be adopted and last to be abandoned. It has been honored by a distinguished name of which it has proved itself worthy, signifying in the Greek, flesh preserver.

The London and Paris Pharmacopœias give the following mode of its preparation : Distil wood tar in an iron retort until white vapors appear ; collect the heavy, oily matter which forms the lower layer of the product, wash it with water slightly acidulated with sulphuric acid ; then distil it in a glass retort, rejecting the first portions, which are chiefly euopione, and heat the product with a solution of potassa sp. gr. 1, 12, shaking the mixture strongly. When it has settled pour off the layer of euopoine, from the surface and expose the combined potash and creosote to the air until it becomes black, then saturate with dilute sulphuric acid, pour away the watery liquid and distil the product in glass. Repeat the treatment by exposure, potash, sulphuric acid and distillation three times or oftener, until the combination of creosote and potash ceases to become colored by the action of the air, then saturate it with concentrated phosphoric acid, and distil the creosote, rejecting the first portions.

This drug, when obtained in its purity, is one of our most valuable remedies. In connection with the above-named remedy, and before I proceed to give its medical properties, I may introduce carbolic acid, as these two substances so closely resemble each other in appearance, odor, and general properties—the one being a distillation of wood-tar, and the other a distillation of coal-tar. Their therapeutical properties are similar, and may therefore be given together.

They are both antiseptics, stimulants, styptics irritant and escharotics, and to creosote some add that of nervine. They both unite with albumen and gelatine, forming with them insoluble compounds. They possess the property of promoting healthy granulations and hastening the healing process. They relieve pain without causing inflammation. They are valuable agents in arresting the process of supuration. They are considered very efficient in arresting, temporarily, the pain arising from an exposed pulp, and also in rendering a cavity less sensitive. Some good practitioners are in the habit of washing out the cavity with creosote immediately prior to filling. Many other uses will suggest themselves to the dental practitioner. Indeed, it is considered indispensable to our success. The great importance of this medicine will justify the time and attention that has been bestowed upon it in this essay.

Your attention will next be called to that important class of medicines, astringents proper. Although the two preceding possess this property in common with others, yet it is not their distinguishing characteristic. Astringents are few and entirely distinct in their mode of operation from all others. They do not necessarily act in the blood, although many haematics are also astringents. They do not pass from the blood to the nerves. As neurotics act directly on nerves, so these act directly and especially on muscular tissue or fiber. They cause this to contract, whether it be striped and voluntary, or of the involuntary and unstriped kind. Taken into the blood in a state of solution, they pass through the walls of the capillaries to the muscular tissue.

The greatest and most valuable of all the vegetable astringents is Tannin—Acidum Tannicum. With this astringent at hand we could almost afford to dispense with all others. This remedy is obtained by causing commercial sulphuric ether to percolate through pounded galls in a glass adopter, closed at the lower end with carded cotton. The liquor obtained separates in two parts; pour off the upper layer and evaporate the lower portion with a moderate heat to dryness.

This medicine is purely an astringent. Its principal use in dentistry is as a styptic in hemorrhage, relaxation of the uvula, and in chronic inflammation of the fauces. It, like creosote and carbolic acid, combines with albumen, fibrin, and gelatin, and forms in this case an insoluble tanate, thus preserving the parts beneath from the influence of irritating agents, till the case has time to terminate in resolution.

Having considered the principal vegetable astringent as the type of the class, it may now be proper to take up the principal metalic astringent or styptic, which I take to be Ferri Chloridum. What tannin is in the vegetable kingdom, ferri chloridum is in the mineral kingdom. With these two agents in our possession we needhardly encumber our office with any others.

The proto-chloride is made by dissolving clean iron turnings in muriatic acid, boiling the solution on excess of iron, decanting as soon as settled, and evaporating quickly to dryness. The perchloride is made by evaporating to dryness a solution of red oxide of iron in muriatic acid. I need only say that this is a powerful astringent and styptic, and may be used in place of the tannin for these purposes if desired.

Acidum Sulphuricum is of sp. gr. 1,845. This acid with its officinal arromaticum has come extensively into use by dentists in the treatment of diseased osseous and dentine structures, as well as in alveolar abscess. The elixir of vitriol is composed of the following proportions: Sulphuric acid, f.ziii. ss; ginger bruised, zi; cinnamon, zi. ss; Alcohol, oil. Digest the alcohol and acid together for three days, then add the ginger and cinnamon and macerate for a week; lastly, filter through paper.

In general practice it is used as an astringent, tonic and refrigerant, but in dental practice it is depended upon to break up and remove unhealthy bone and dentine, dental calculi, and destroy indolent ulcers. Such is its affinity for lime salts, that it should be used with great caution.

Oxalic Acid, the great bleaching agent, has been highly recommended for bleaching teeth. This, under certain circumstances, may prove a very efficient agent, especially when the discoloration is produced by the salts of iron. A tooth congested with red blood globules may be very properly created with this agent; but it must be remembered that this acid has a great affinity for lime salts, and therefore must not remain long in contact with tooth substance.

Argenti Nitras, Lunar Costic, was formerly and perhaps is to some extent yet, used by the dental profession. It is obtained by dissolving ℥i. of silver in a mixture of f.℥v. of nitric acid, f.℥ii of distilled water on a sand bath. Evaporate the solution to dryness, fuse and pour into moulds. *Medical properties*, tonic, antispasmodic, applied externally, it is a stimulant and escharotic. Like sulphate of copper, sulphate of zinc and acetate of lead, it forms a coagulum with albumen, but the clot is soluble in an excess of albumen, while that formed by tannic acid, perchloride and persulphate of iron is not soluble in albumen or any other constituent of the blood. It is a powerful caustic when applied to the soft parts, or to the bony tissues. It acts upon the gelatinous portions of the tooth, destroying its vitality to the extent of the combination that takes place. It is an active poison, but can be neuteralized by a strong solution of common salts, which converts it into chloride of silver.

There is a class of medicines termed neurotics, from neuron, a nerve. This class of medicines is temporary in its action. They influence the nervous system, exciting it, depressing it, or otherwise altering its tone. They are chiefly useful in the temporary emergencies of acute disorders. They can seldom effect a permanent cure, unless when the contingency in which they are administered is also of a temporary nature. Neurotics pass out of the blood to the nerves which they influence. It is further affirmed that they are transitory in their action. They appear to effect molecular changes in the nerve fiber, similar to those by which the phenomena of the senses are produced, and which are by nature transitory in their results. There are three divisions of neurotics. The first set are of use when there is a dangerous deficiency of vital action. These are stimulants. They exalt nervous force, either of the whole nervous system or only of a part of it. A second class, called narcotics, first exalt nervous force and then depress it —they have thus a double action ; but they have also a peculiar influence over the functions of the brain, which is different from any possessed by any nerve medicine. They control the intellectual part of the brain as distinguished from its organic functions; the powers of *mind* more than those of *life*. Some narcotics tend to produce inebriation, others sleep, others again delirium. Again, some neurotics tend simply and primarily to depress nervous force, and are said to have a sedative effect; but to have any permanent effect they must be constantly repeated. Neurotics may be further considered under three general divisions, viz: 1st. Narcotics or cerebral, such as opium, prussic acid, alcohol, chloroform, camphor, tobacco, coculus indicus, &c. 2nd. Spinal, including nux vomica, strychnia, &c. Then there is a third class called cerebro-spinal, including hemlock, monk's hood or aconite, belladonna, lobelia, digitalis, &c. The action of these classes is indicated by their names or classification. The cerebral acting on the cerebrum, the spinal acting on the spinal cord, and the cerebro-spinal on both cerebrum and spinal cord.

For some reason, no doubt good and sufficient, the dental profession has shown a decided preference for that class of neurotics called cerebro-spinal, nearly to the exclusion of all others. It is most probable that these cerebro-spinal agents, such as aconite, is selected for its known properties or powers to affect the nerves of sensation, by exciting, depressing, or destroying sensibility. These neurotics are almost invariably virulent poisons, and must be used with great care and skill. Dentists use these drugs externally to an inflamed nerve, by the use of which they expect to produce sedative influence, to lower the degree of excitability, or perhaps temporarily to paralyze the part. Aconitum and belladonna have been the two principal agents relied on by dentists. The tincture of aconite is sufficiently strong for topical application. The active principle, or alkaloid, should not be employed. Belladonna belongs to the same class of neurotics and is possessed of similar properties, and must be used with the same caution.

In this essay I have referred to about six or eight medicines that I considered the most prominent and useful in the Dental Materia Medica. The time and space allotted to this essay will not justify the introduction of any more at present. In conclusion, I would call the attention of the members of this association to the fact, that the field of research has been and still is entirely too limited in the department of Dental Materia Medica. The mechanical department has made great advancement, producing many invaluable improvements; but the scientific department has been sadly neglected. Science should keep pace with art. Educated men occupying favorable situations in large cities, where there are opportunities to experiment, should investigate the properties of the untried neurotics, in hope that that some latent power might be brought forth which would be of great advantage to the dental profession. There is at present great diversity of opinion existing among our leading men on the treatment of dental disease, each one advancing a theory differing from all others. Each new theory runs its course in a short time and is abandoned to give place to another. This state of things goes to show that our theories are only in their formative state, and need time, sound judgment and scientific research to settle upon some sound and uniform method of practice. Theories, crude and unproved, are set forth with too much confidence, and adopted by many, only to be abandoned as worthless, or perhaps injurious, after a short trial. Judging by the progress that dentistry has made in the past quarter of a century, we have reason to be encouraged. The light of a brighter day is dawning when dentistry will stand pre-eminent among its sister professions.—*Lake Erie Dental Association—Penn. Journal of Dental Science.*

